# Hospital service quality based on HEALTHQUAL model and trusting nurses at Iranian university and non-university hospitals: a comparative study

**DOI:** 10.1186/s12912-020-00513-y

**Published:** 2020-12-10

**Authors:** Reza Nemati, Masoud Bahreini, Shahnaz Pouladi, Kamran Mirzaei, Farkhondeh Mehboodi

**Affiliations:** 1 Nursing and Midwifery School, Bushehr University of Medical Sciences, Bushehr, Iran; 2 Medical School, Bushehr University of Medical Sciences, Bushehr, Iran

**Keywords:** Quality, Services, Patient satisfaction, Trust, Hospital

## Abstract

**Background:**

Establishment and improvement of patients’ trust in healthcare organizations like hospitals necessitate delivery of high-quality services by nurses, as the largest group of healthcare providers. The present study aimed to compare hospital service quality based on the HEALTHQUAL model and trusting nurses at university and non-university hospitals in Iran.

**Methods:**

This comparative cross-sectional study was conducted on 990 patients admitted to university and non-university hospitals located in Bushehr Province, southern Iran, who were selected using the stratified random sampling method. The data were collected through the HEALTHQUAL questionnaire and the Trust in Nurses Scale, and then analyzed via the SPSS Statistics software (version 22) as well as the General Linear Model (GLM) univariate procedure and the Chi-square test with a significance level of 0.05.

**Results:**

The study findings revealed that the mean values of real quality (perceptions) and ideal quality (expectations) were 3.89 ± 0.69 and 4.55 ± 0.47, respectively. The gap between the real and ideal quality (− 0.64) was also larger at non-university hospitals from the patients’ viewpoints. Comparing various dimensions of service quality, the largest gap at university and non-university hospitals was associated with “environment” (− 0.13) and “empathy” (− 0.18), respectively. Additionally, the mean scores of the patient trust in nurses at university and non-university hospitals were 10.34 ± 5.81 and 8.71 ± 4.05, respectively, being a statistically significant difference (*p* <  0.001).

**Conclusion:**

The study results demonstrated that hospital service quality and trusting in nurses were at higher levels at the university hospital than the non-university one; however, hospital service quality was at a lower level than what the patients had expected. Accordingly, hospital managers and policy-makers were suggested to focus on patients to reduce gaps in service quality, to promote service quality, and to provide better healthcare services to patients.

**Supplementary Information:**

The online version contains supplementary material available at 10.1186/s12912-020-00513-y.

## Background

After Iran’s considerable progress in providing primary health services, in recent years, improving the quality of health services in hospitals has been regarded by policy-makers and managers. In Iran, university and non-university hospitals provide their non-stop health services to patients [1]. As one of the organizations providing continuous healthcare services, these hospitals have a special position in the health system [2]. Therefore, service quality, regardless of whether hospitals are university-based or not and they are administered by which institutions or organizations, needs to be evaluated [3]. Hospital service quality can lead to employee and patient satisfaction as well as loyalty in patients toward hospitals, and encourages them to recommend such healthcare centers to friends and acquaintances [4, 5]. Consideration of quality can thus reduce costs in organizations, increase efficiency, and provide long-term stability [6]. Accordingly, improved levels of satisfaction in patients as the frequent customers of hospitals are a fundamental objective by the managers and healthcare providers (HCPs) of such centers [7].

Despite all developments, challenges in delivery of quality health care still exist. Unfortunately, medical errors and their adverse effects cast doubt on service quality and cause patients to lose trust in such services [8]. Recent studies have estimated that medical errors are the third leading cause of death in the United States [9]. The results of a study conducted in a university hospital in Iran had reported 158 cases of medical errors within one year [10]. In addition, costs related to healthcare services and their quality improvement put relentless pressure on customers and governments. Some statistics indicated that approximately $7.8 trillion were expended on healthcare services in 2017 [11]. In Iran, 6.89% of the Gross Domestic Product (GDP) equal to $350 per case was allocated to this sector in 2014 [12].

Regarding advances in different communities and higher levels of awareness, individuals develop higher expectations of receiving healthcare services, and patients admitted to hospitals demand high-quality ones. Based on this public demand, improvements in existing practices for effective management of healthcare services have been made more remarkably [13]. In terms of healthcare services, attention to patients’ viewpoints has turned into the milestone of service quality [14]. Thus, evaluation of such attitudes toward the quality of healthcare services is one of the principles of progress and success in any healthcare facility [15]. In this respect, different instruments have been developed to evaluate service quality. Researchers have widely considered SERVQUAL as one of these instruments. However, SERVQUAL faces challenges in evaluating the quality of health services. This instrument was developed beyond the health system for general services, not considering the main dimensions of the quality of healthcare services such as availability, affordability, caring, and medical outcomes. For this reason, the HEALTHQUAL instrument has been introduced as a substitute for this tool in evaluating the quality of health services [16].

Enhanced service quality and increased levels of satisfaction can ultimately boost customers’ trust in organizations [4]. Trust can thus improve patients’ mental well-being and peace of mind. Since human needs continually expand, mere attention to the subjective dimensions of welfare is not the way to achieve satisfaction, but consideration of individuals’ mental needs, especially patients’ ones is extremely important [17, 18]. Therefore, establishment of patients’ trust in hospitals and healthcare services has been included on the agenda of healthcare policymakers [19]. Trust in hospitals can be built through strong and long-term interactions between patients and their families and HCPs, and it remains relatively stable [17]. The relationships between patients and their families and hospitals and nurses are very extensive; therefore, patients’ expectations of nurses are far higher compared to other employees and they typically demand high-quality services [20]. Trust is the most important factor in the formation of relationships between patients and nurses, with numerous positive effects on improving patients and increasing the treatment effectiveness [21].

Many studies measuring hospital service quality and patients’ trust in nurses have thus far reflected challenges and defects in this field. For example, in a meta-analysis evaluating the quality of healthcare services based on the SERVQUAL model in Iran, a negative gap was reported in 12 provinces surveyed [22]. In another study, using the SERVQUAL model to measure service quality in healthcare facilities, a significant difference was observed in the real and ideal quality of services in Tehran, as the capital city of Iran [23]. In Pakistan bordering Iran, the results of a survey indicated insufficient delivery of services to patients due to the small number of personnel at public hospitals. Moreover, patients’ perspectives revealed their dissatisfaction with service quality [24]. Some scientific evidence further demonstrated that the quality of healthcare services affected the patient trust. Researchers thus concluded that trusting in nurses was an important and complex phenomenon demanding nurses to develop some professional characteristics and reinforce them [25]. Lien et al. (2014) also underlined the positive impact of service quality on patients’ trust in hospitals [26].

A review of previous studies indicates that the focus in most studies has been solely on evaluating service quality in special centers with no comparative approach. Moreover, the majority of the studies have been conducted via the SERVQUAL model as a non-specific quality measurement instrument with some limitations to measure the quality of healthcare services. As university and non-university hospitals are different in terms of management, human resources, levels of education, and policies, several questions always rise on the quality of healthcare services provided and levels of public trust in such centers; this requires further research.

### Aim of the study

The present study aimed to compare hospital service quality based on the HEALTQUAL model and trusting in nurses at university and non-university hospitals in Iran.

## Methods

### Design, settings and participants

This comparative cross-sectional study was conducted on patients admitted to two major university and non-university hospitals. These hospitals had 350 and 320 beds, respectively, and they were known as the main centers providing medical specialty and subspecialty services in Bushehr Province, southern Iran.

These two hospitals were selected, since they were the main referral centers for patients in Bushehr Province and were very similar in terms of number of wards, number of beds and type of health services provided. The patients were selected using the stratified random sampling method. The sample size was determined according to the number of beds in each hospital, number of beds in each ward, and the whole sample required in this study. With reference to the study conducted by Mossadegh-Rad [27] and the total quality ratio of the university hospital by 0.85, assuming an acceptable difference of 0.1 between university and non-university hospitals as well as α = 0.05 and β = 0.2, using the sample size formula

$$ n=\frac{Z_{1-\alpha 1z}\sqrt{P\left(1-P\right)}+{Z}_{1-\beta}\sqrt{P_0\left(1-{P}_0\right)+{P}_1\left(1-{P}_1\right)}}{{\left({P}_0-{P}_1\right)}^2} $$

and comparison between wards using the correction formula $N=N\times \sqrt{K}$, the sample size in each hospital was calculated to be 410 persons. Nevertheless, 1000 patients finally received the questionnaires due to the possibility of incomplete cases. In line with the survey conducted by Charalambous et al. [28], considering the trust score of nurses and the differences in the comparison group by d = 1, the previously calculated sample size could cover the trust score difference among nurses.

### Eligible criteria

The inclusion criteria in this study were at least 24 h after admission, written patient consent to participate in research, ability to respond to the questionnaires, consciousness, and no psychiatric disorders based on self-reporting. According to the inclusion criteria, the pediatric and maternity wards as well as the Intensive Care Units (ICUs) wherein the patients were unable to complete the questionnaires, were excluded. Therefore, the patients admitted to emergency departments for adults, women’s wards, surgical wards for women, internal wards for women, surgical wards for men, internal wards for men, oncology wards, and Coronary Care Units (CCUs) were included. The study samples were selected from the patients admitted to the above-mentioned wards within all three shifts. The exclusion criteria were unwillingness to continue the research and incomplete questionnaires. The patients also became informed of the voluntary basis of the study, and they were assured that their personal information and names would not be entered into the questionnaires to respect the confidentiality policy.

### Data quality control

Four data collectors were recruited from postgraduate nursing students. These students were not the staff of the two selected hospitals. To ensure accuracy, consistency and completeness, one member of the research team closely supervised the data collection process. Before data collection, in a session, the chief researcher introduced the data collectors to the questionnaires and the data collection process.

### Data collection procedure and instruments

Data were collected from September to December 2018. Questionnaires were directly distributed among the patients by the data collectors and were delivered to them upon completion. For each participant, it took approximately 10 to15 minutes to complete the questionnaires. To collect the data, a demographic characteristics information form, the HEALTHQUAL questionnaire, and the Trust in Nurse Scale were used.

### HEALTHQUAL

The HEALTHQUAL questionnaire was designed by Mossadegh-Rad (2018) to measure the quality of healthcare services using 30 items evaluating customers’ perceptions and expectations within four dimensions of “environment” (11 item), “empathy” (12 items), “efficiency” (3 items), and “effectiveness” (4 items) (Table 1). All of the items could evaluate patients’ viewpoints on service quality in two parts, i.e., patients’ perceptions (real quality) and patients’ expectations (ideal quality). The options for each item were also set on a five-point Likert scale. In addition, a four-part item could assess the “importance” of the dimensions of service quality. In this questionnaire, the quality gap was equal to the difference between expectations and perceptions multiplied by importance. Accordingly, a negative gap indicated unacceptable quality, a zero gap represented acceptable quality, and a positive gap showed quality beyond customers’ expectations. Scores 1–1.80, 1.81–2.60, 2.61–3.40, and 3.41–4.20 denoted very poor, poor, moderate, and good service quality, respectively. Additionally, scores above 4.20 specified very good service quality. In the study conducted by Mossadegh-Rad, the Cronbach’s alpha coefficient to endorse the reliability of the HEALTHQUAL questionnaire was determined to be 0.94. The content validity of the given questionnaire was similarly confirmed with the content validity index (CVI) =0.71 and the content validity ratio (CVR) =0.71 [27].

**Table 1:** Domain of HEALTHQUAL

Domain	Description	Items
Environment	Physical facilities, buildings, equipment, and HCPs.	11
Empathy	Interactions between HCPs and recipients, containing characteristics such as respect, courtesy, humility, empathy, help, and accountability.	12
Efficiency	Optimal use of resources, represented cost-outcome ratio, and involved factors such as waiting time, speed of service delivery, and value of services received for costs paid.	3
Effectiveness	Meeting the goals of customers (patients), incorporating safety and comprehensiveness of services received, pain relief, and expected health	4
Efficacy	The extent to which HCPs could achieve their goals.	

### Trust in Nurses Scale

The Trust in Nurses Scale was developed by Radwin and Cabral (2010) to measure patients’ trust in nurses. It contained five items, each one with six options, namely, 1 = never, 2 = rarely, 3 = some of the times, 4 = often, 5 = usually, and 6 = always. Accordingly, higher scores showed higher levels of trust among patients. The content validity of this instrument was carried out recruiting a panel of experts by CVI = 0.90. Furthermore, the reliability of the questionnaire concerned was determined via internal consistency with the Cronbach’s alpha coefficient of 0.93 [29].

### Ethical consideration

This study was approved by the Ethics Committee of Bushehr University of Medical Sciences, Bushehr, Iran, under the code of ethics: IR.BPUMS.REC.1397.054. It was also implemented by observing ethical considerations, including obtaining informed consent and acting in accordance with the principles of confidentiality and privacy.

### Data analysis

To analyze the data, the SPSS Statistics software (version 22) was employed. Descriptive statistics (frequency and frequency percentage, mean and standard deviation), text narration, tables and a figure were used to present results. For inferential statistics, Chi-square test, independent-samples t-test, and the univariate general linear model (GLM) were used. For all cases, values less than 0.05 were considered statistically significant.

## Results

From 1160 patients who were asked to participate in the study, 160 patients declined to participate in the study, and 10 patients excluded from the study due to incomplete questionnaires. Finally, A total of 990 patients were included in our study (response rate = 99%). The number of female participants from both university and non-university hospitals was slightly higher than that of males with 265 (53.50%) and 290 patients (58.60%), respectively. Moreover, married participants with 322 (65.10%) and 334 cases (67.50%) constituted the majority at university and non-university hospitals, respectively. The patients’ length of stay at university and non-university hospitals was 4.21 (4.03) and 4.47 (5.37) days, respectively. In terms of level of education, the bulk of the patients referred to both hospitals had diploma. As a whole, the patients admitted to both hospitals were significantly different in terms of levels of education, occupation, levels of income, and types of insurance coverage (Table 2).

**Table 2:** Summary of demographic characteristics of patients in university and non-university hospitals

Variables	Subgroup	Hospital	Number	Frequency (%)	*P* Value*
**Education**	Illiterate	University	138	28	0.001
		Non-University	102	21	
	Middle School degree	University	84	17	
		Non-University	106	21	
	Diploma	University	160	32	
		Non-University	163	33	
	Associate Degree	University	31	6	
		Non-University	58	12	
	Bachelor and higher	University	82	17	
		Non-University	66	13	
**Job**	Unemployed	University	245	49	< 0.001
		Non-University	228	46	
	Employed	University	236	48	
		Non-University	190	38	
	Retired	University	14	3	
		Non-University	77	16	
**Income**	Less than $50	University	150	31	< 0.001
		Non-University	99	20	
	$ 50 to $100	University	108	22	
		Non-University	125	24	
	$ 100 to $200	University	151	31	
		Non-University	193	39	
	Over $ 200	University	78	16	
		Non-University	86	17	
**Ward**	Women surgery	University	60	12	< 0.001
		Non-University	70	14	
	Male Internal	University	59	12	
		Non-University	73	15	
	Female Internal	University	58	12	
		Non-University	85	17	
	Men’s Surgery	University	98	20	
		Non-University	57	12	
	Gynecological	University	59	12	
		Non-University	70	14	
	Hematology and Oncology	University	31	6	
		Non-University	53	11	
	Emergency	University	85	17	
		Non-University	66	13	
	CCU	University	45	9	
		Non-University	21	4	
**Insurance**	No insurance	University	80	16	< 0.001
		Non-University	16	3	
	Health insurance	University	144	29	
		Non-University	78	16	
	Tamin-Ejtemayi Insurance	University	168	34	
		Non-University	304	61	
	Insured by Armed Forces	University	30	6	
		Non-University	57	12	
	Others	University	73	15	
		Non-University	40	8	

*Chi Square Test

**Fig. 1:**
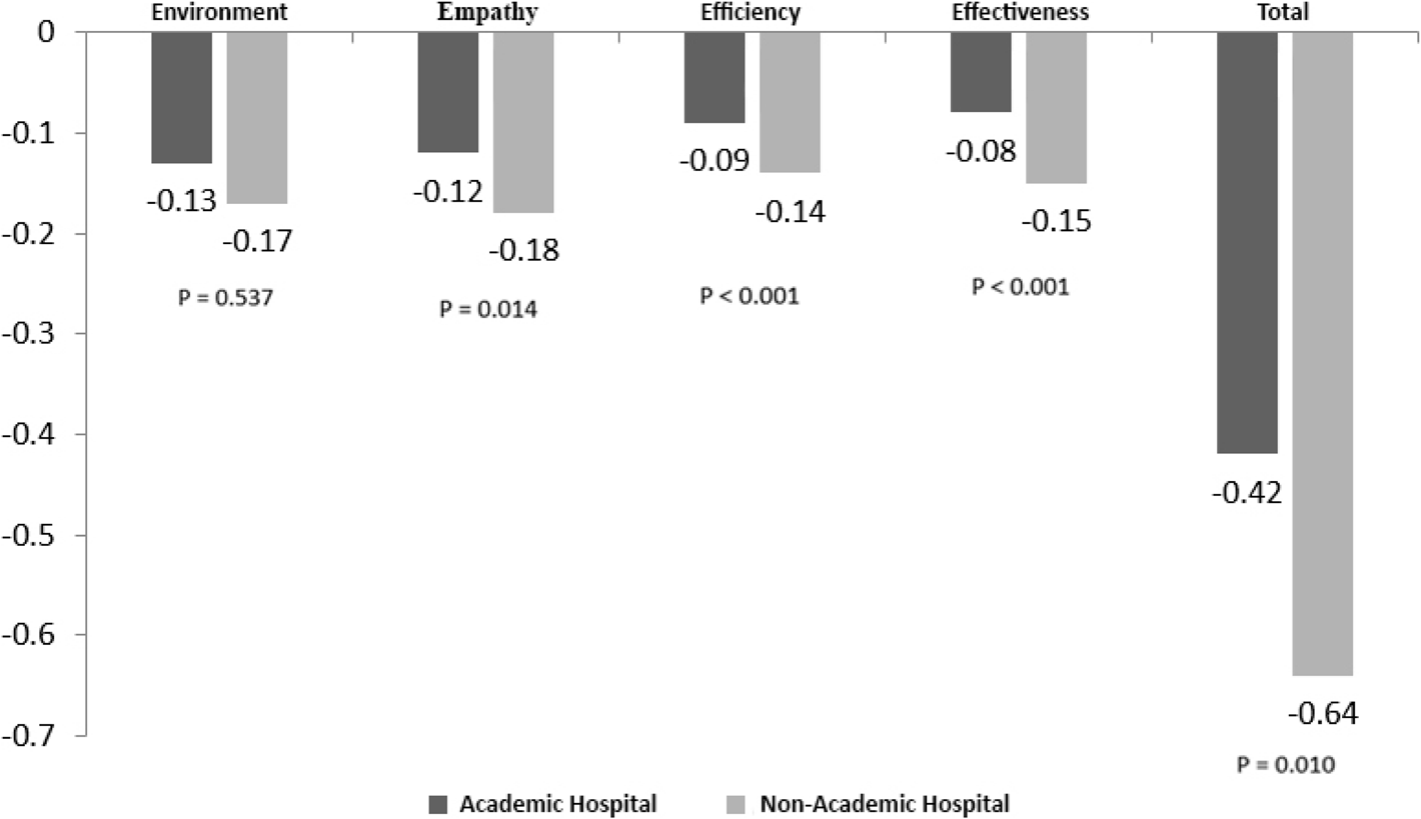
Comparison of service quality gap between university and non-university hospitals

The study results revealed that the patients at both university and non-university hospitals had considered the highest and the lowest quality of services provided in the real situation (perceptions) regarding the dimensions of “empathy” and “efficiency”, respectively. Other results demonstrated that, while most patients at the university hospital had expectations of service quality associated with “empathy”, those at the non-university hospital had introduced the dimension of “environment”. The patients at both hospitals had the lowest expectations of service quality concerned with “efficiency”. In addition, among the various dimensions of the quality of services provided, the university-hospital patients gave more and less importance to “empathy” and “effectiveness”, respectively, whereas patients at non-university hospitals paid more attention to “environment” and gave the least importance to “efficiency” (Table 3). Other results of the study revealed that the highest and the lowest gaps in service quality at the university hospital were related to the dimensions of “environment” (− 0.13) and “effectiveness” (− 0.08), respectively, while the highest and the lowest service-quality gaps at the non-university hospital were associated with the dimensions of “empathy” (− 0.18) and “efficiency” (− 0.14) (*p* <  0.001), respectively. In a general comparison of both hospitals, it became evident that expectations and perceptions of service quality among the patients referred to the non-university hospital were at higher levels than those among the patients referred to the university hospital. Moreover, the gap amount in service quality at the non-university hospital (− 0.64) compared to that at the university one (− 0.42) was larger (*p* = 0.01) (Fig. 1). The patients’ trust in nurses at both hospitals also showed a significant difference; in other words, trusting in nurses was higher at the university hospital than at the non-university one (Table 3).

**Table 3:** Determining and comparing the average perceptions, expectations, importance and trusting nurses in university and non-university hospitals

Quality of Service	Dimensions	University Hospital M ± SD	Non-University Hospital M ± SD	***P*** Value
**Perceptions**	Environment	3.57 ± 0.93	3.90 ± 0.72	< 0.001
	Empathy	3.62 ± 0.89	3.92 ± 0.74	< 0.001
	Efficiency	3.48 ± 0.80	3.82 ± 0.76	< 0.001
	Effectiveness	3.56 ± 0.81	3.91 ± 0.72	< 0.001
	Total	3.54 ± 0.77	3.89 ± 0.62	< 0.001
**Expectations**	Environment	4.07 ± 0.95	4.61 ± 0.52	< 0.001
	Empathy	4.07 ± 1.03	4.55 ± 0.51	< 0.001
	Efficiency	3.87 ± 1.19	4.47 ± 0.62	< 0.001
	Effectiveness	3.95 ± 1.10	4.57 ± 0.50	< 0.001
	Total	4.02 ± 0.94	4.55 ± 0.47	< 0.001
**Importance**	Environment	0.26 ± 0.04	0.25 ± 0.02	< 0.001
	Empathy	0.27 ± 0.05	0.29 ± 0.02	< 0.001
	Efficiency	0.24 ± 0.05	0.22 ± 0.03	< 0.001
	Effectiveness	0.23 ± 0.05	0.24 ± 0.02	< 0.001
	Total	1.00	1.00	–
**Trusting Nurses**	–	10.34 ± 5.81	8.71 ± 4.05	< 0.001

## Discussion

This study aimed to compare hospital service quality based on the HEALTHQUAL model and patients’ trust in nurses at the selected university and non-university hospitals in Iran. The study results indicated that the patients’ perceptions and expectations of service quality were higher at the non-university hospital than at the university one. The findings of one survey comparing the quality of services provided at public and private hospitals in Ghana were also consistent with the present study results [30]. Unquestionably, these findings could demonstrate the higher service quality at the non-university hospital, but several factors might have also affected patients’ perceptions and expectations of service quality. For example, it appears that the socioeconomic status of patients referred to different hospitals should be regarded in analyzing the results of perceptions and expectations assessments. For example, the non-university hospital examined in this study had more expensive and better insurance coverage. On the contrary, upon admission, small costs could be charged from patients in return for services. This could thus shape the perceptions of hospitalized patients and improve their levels of satisfaction. Nevertheless, it is possible that patients’ expectations of service quality are also influenced by another aspect of their health insurance. In fact, these patients expect to receive more services, since they pay considerable premiums on a monthly basis for their insurance coverage.

Furthermore, the study results showed that the gap in service quality at both university and non-university hospitals was negative, but such a gap in all dimensions of service quality at the non-university hospital was higher. It is interesting that while the patients’ perceptions of the real situation at the non-university hospital were better, they had failed to meet their expectations and caused a gap in service quality. From this perspective, it appears that the patients admitted to the university hospital were more satisfied than those referred to the non-university one were. However, the results of a study recently conducted in Saudi Arabia neighboring Iran demonstrated a smaller gap in service quality by non-governmental hospitals, and patients’ satisfaction with such hospitals were also at higher levels [31]. However, unlike Iran, most hospitals in Saudi Arabia are non-university and private ones, and the competitive nature of these centers can give them more incentives to deliver high-quality services to patients and win their satisfaction, since managers of such hospitals have to focus on the improvement of service quality to gain more profits.

Not surprisingly, the results of most studies conducted in Iran and other countries have further established a negative gap in the quality of services provided by hospitals [14, 32–34]. The fact is that customers of medical centers are not physically and mentally well, once they are admitted. Patients’ sufferings and pains from diseases can accordingly affect their perceptions and judgments; therefore, it is not easy to satisfy them, and it is even a difficult task to reach a positive gap in service quality. However, considerable results have been reported in a small number of studies, e.g. one survey on one of the hospitals in southeastern Iran noted that patients showed their satisfaction with the quality of healthcare services and the gap was positive [35]. The results of a study in Spain presented a positive gap in service quality in the dimensions of “environment” and “empathy” [36]. Surveying the participants in these two studies, the results might not be surprising. The study setting was a field hospital in southeastern Iran as a temporary base to meet the emergency needs of the target population residing there. Accordingly, factors such as dire needs and low levels of socioeconomic variables in the region concerned might have led to lower levels of expectations and positive assessments by patients regarding service quality. The study in Spain also recruited a unique statistical population, i.e., patients with high levels of consciousness admitted to ICUs. Thus, the positive gap in the dimensions of “environment” and “empathy” could be justified with respect to the availability of facilities and appropriate equipment as well as the larger number of staff working in this special ward.

Regarding the comparison of the patients’ trust at both hospitals concerned, the results revealed that the patients had more trusted university-hospital nurses than those at the non-university hospital. In general, despite some shortcomings in the facilities of public and university hospitals, the patients’ trust in HCPs in these centers appeared to be at an acceptable level. The results of one survey investigating the relationship between quality of healthcare services and patient satisfaction at university hospitals in Iran established that trust in nurses among patients was above average [37]. The results of another study at a university hospital also indicated that only 5% of the patients had little trust in nurses [38]. These findings confirmed the results of the present study, demonstrating that the patients placed more trust in nurses at university hospitals than in those at non-university ones. A noteworthy point in this line is the crucial role of “empathy”. Given that university hospitals had a smaller gap in service quality in terms of “empathy” compared to non-university ones, achieving such results was expected. In addition, it appears that academic atmosphere and presence of teachers and students in clinical wards were among factors building more trust in nurses among patients, which has been thus far confirmed by some scientific evidence [39].

### Strength and limitations

This study was conducted using a relatively large sample size, which can be one of its strengths. At the same time, there were several limitations. The results of this study, however, were related to two hospitals with special locations and facilities, which should be considered in generalizing these results. Another important limitation of this research is the use of a questionnaire, which due to its inherent limitations, is only an incomplete tool to collect information, and the conversion of qualities into quantities (questionnaire options) usually limits the generalization of fieldwork results. The present study is no exception to this rule. Therefore, it is suggested that in future studies, a qualitative approach be used to evaluate the quality of services. In addition, in the present study, the quality measurement of services was based only on receiving the opinions of patients, and the opinions of other stakeholders such as doctors, nurses, managers and other service providers were not considered. It is suggested that in future research, the quality of services perceived by patients and the quality of services perceived from the perspective of employees be compared. Combination of two internal and external organizational attitudes toward quality can provide managers with a better picture of patient satisfaction.

## Conclusions

In this study, a negative gap was observed at both university and non-university hospitals in all dimensions of service quality. The results indicated that, although satisfaction with the real situation at the non-university hospital was higher than that at the university one, expectations of the patients admitted to the non-university hospital were at a higher level, causing a more negative gap in the service quality of the non-university one. However, this study revealed that patients’ trust in nurses at the university hospital was higher than that among patients referred to the non-university one.

As trust in patient compliance with care regimen can be effective, upgrading this component should be put in the agenda by hospital managers. The study results also imply that elimination of existing gaps requires further efforts by hospital managers and policy-makers to improve service quality in all dimensions. A better understanding of factors affecting dissatisfaction, lack of trust, and gaps in the quality of healthcare services can be accordingly employed as the first and foremost step in the promotion process. Moreover, qualitative research appears to provide a better and deeper understanding of the viewpoints and experiences of patients admitted to different hospitals regarding the concepts of “service-quality” and “trust”.

## Supplementary Information

**Additional file 1.** Hospital Service Quality Scale.

**Additional file 2.** Trust in Nurses Scale.

## Data Availability

The datasets used and/or analyzed during the current study are available from the corresponding author on reasonable request.

## Abbreviations

HCPs: Healthcare Providers
GLM: General Linear Model
GDP: Gross Domestic Product
ICU: Intensive Care Unit
CCU: Coronary Care Unit
SERVQUAL: Service Quality
HEALTHQUAL: Health Quality
