# Supplementary material for: Hospital service quality based on HEALTHQUAL model and trusting nurses at Iranian university and non-university hospitals: a comparative study
Source: BMC Nurs. 2020 Dec 10;19:118. doi: 10.1186/s12912-020-00513-y (PMC7731503; doi:10.1186/s12912-020-00513-y)
Supplement: Supplementary file 1 — Additional file 1. Hospital Service Quality Scale. [file 12912_2020_513_MOESM1_ESM.pdf]

Hospital Service Quality Scale

No.	Item	How important I think it is (my expectation)					What I experienced in this hospital				
		Unimportant	Slightly Unimportant	Slightly important	Important	Very Important	Very Bad	Bad	Average	Good	Excellent
1	Easy and fast access to the hospital and the needed wards and specialties										
2	Clean hospital environment (patient rooms, waiting area, sanitary facilities, etc.)										
3	Feeling comfortable in the hospital (sufficient space, comfortable beds, no noise, etc.)										
4	Quality, quantity, cleanliness, and proper distribution of clothes and sheets										
5	Quality, quantity, cleanliness, and proper distribution of hospital food										
6	Easy and timely access to needed medications										
7	Access to entertainment and appliances (TV, newspaper, magazine, refrigerator, etc.)										
8	Modern and advanced diagnostic and therapeutic equipment										
9	Easy and fast access to clean, orderly, and disciplined staff when needed										
10	High experience and expertise of hospital staff, including doctors, nurses, etc.										
11	Clarity and comprehensibility of hospital instructions and brochures										
12	Behavior and attitude of hospital staff in terms of being respectful and being committed to their job										
13	Hospital staff being trustworthy and making people feel comfortable to communicate with them										
14	Staff being compassionate and patient and spending time to help patients and address their problems										
15	Staff being understanding of patients' individual needs and expectations and being flexible in meeting them										
16	Staff paying attention to patients as human beings and respecting their opinions, requests, and expectations										
17	Staff being respectful of patients' independence and private space and their medical secrets										
18	The ability of staff to convey accurate information to admitted patients in a comprehensible way										
19	The ability of staff to educate patients (about illness, self-care, post-discharge care, etc.)										
20	Staff making it possible for patients to participate in the treatment program; Staff considering the opinions of patients in the program										
21	Staff behaving respectfully toward patients' guardians and visitors; Staff making it possible for patient guardians to participate in the treatment program										
22	Staff providing the necessary guidance and instructions to patients during the discharge process										
23	Cooperation and teamwork among the staff of different wards, especially between medical and laboratory wards										
24	The value of the services received for the cost paid										
25	Speed of hospital services (fast admission, fast provision of medical, nursing, and laboratory services)										
26	Short waiting time and minimum delay in services (providing services according to the planned schedule)										
27	Achieving the expected results with acceptable outcomes in terms of improvement in physical and mental health										
28	Relief from pain										

No.	Item	How important I think it is (my expectation)					What I experienced in this hospital				
		Unimportant	Slightly Unimportant	Slightly important	Important	Very Important	Very Bad	Bad	Average	Good	Excellent
29	Receiving no harm in the hospital (safety in providing medical, nursing and laboratory services)										
30	Receiving complete and comprehensive services (referral of the patient to other wards and specialties if needed)										
