# Supplementary material for: Hospital service quality based on HEALTHQUAL model and trusting nurses at Iranian university and non-university hospitals: a comparative study
Source: BMC Nurs. 2020 Dec 10;19:118. doi: 10.1186/s12912-020-00513-y (PMC7731503; doi:10.1186/s12912-020-00513-y)
Supplement: Supplementary file 2 — Additional file 2. Trust in Nurses Scale. [file 12912_2020_513_MOESM2_ESM.pdf]

Trust in Nurses Scale

No.	Item	Never	Rarely	Some of the times	a good bit of the time	Usually	always
1	How often were your nurses there when you needed them?						
2	How often did you believe that your nurses were acting in your best interest?						
3	How often did you trust what your nurses told you?						
4	How often did your nurses do what they said they would do?						
5	How often did your nurses provide accurate information about the cancer?						
